# Bridging Bone Health: Osteoporosis Disparities in the Rio Grande Valley

**DOI:** 10.7759/cureus.51115

**Published:** 2023-12-26

**Authors:** Ryan P Bialaszewski, John M Gaddis, Blake Martin, Philippe Dentino, John Ronnau

**Affiliations:** 1 School of Medicine, The University of Texas Rio Grande Valley, Edinburg, USA

**Keywords:** osteoporosis, prevention of osteoporosis, racial disparity, health care disparity, rio grande valley

## Abstract

Introduction: Osteoporosis is characterized by decreased bone mass and decreased bone quality, leading to increased bone fragility and risk of fractures. The number of fractures due to osteoporosis is projected to increase to over three million by the year 2025 and cost $25.3 billion annually. It ranks highly among diseases that cause patients to become bedridden with serious complications and reduced quality of life. Additionally, osteoporosis disproportionately affects Hispanics, which comprise most of the Rio Grande Valley (RGV) population. Therefore, our primary objective was to determine the prevalence of osteoporosis within the RGV. Additionally, we had secondary objectives to determine the screening rates of osteoporosis in the RGV and identify other potential risk factors associated with osteoporosis. We hypothesize that individuals residing in the RGV have higher rates of osteoporosis and lower rates of osteoporosis screening than the national average.

Methods: This retrospective observational cross-sectional study utilized Medicare beneficiary data via the "Mapping Medicare Disparities by Population" interactive tool. Osteoporosis data were compared within the RGV (comprising Starr, Hidalgo, Cameron, and Willacy counties) and compared with national averages between the years 2016 and 2021. Statistical analysis included prevalence ratios with 95% confidence intervals and chi-square values when applicable.

Results: Among Medicare beneficiaries residing in the RGV, there are higher rates of osteoporosis compared to the national average (11.5% vs. 7.20%; p < .00001). Screening for osteoporosis within the RGV is above the national average (9.29% vs. 6.67%, p < .00001). Hispanics residing in the RGV have higher overall rates of osteoporosis than Caucasians residing in the RGV (12.3% vs. 8.60%, p < .00001). Females residing in the RGV have nearly twice the rate of osteoporosis compared to the national average (19.1% vs. 11.8%, p < .00001) and 6.58 times the rate of males residing in the RGV (19.1% vs. 2.9%, p < .00001).

Conclusion: Individuals residing in the RGV are disproportionately affected by osteoporosis. Despite increased screening rates seen among Medicare beneficiaries, we also suspect many individuals, uninsured or undocumented, have not received any appropriate osteoporosis screening. Risk factors in the RGV associated with higher rates of osteoporosis could include low education levels, socioeconomic status, physical activity, and mineral intake. These results demonstrate a need to address osteoporosis health literacy, promote earlier interventions to treat osteoporosis and increase healthcare accessibility in the RGV.

## Introduction

Osteoporosis is a common systemic skeletal disorder characterized by low bone mass and microarchitectural deterioration of bone tissue, leading to increased fragility and susceptibility to fractures [1-5]. The World Health Organization defines osteoporosis as a bone mineral density (BMD) that is 2.5 standard deviations or more below the mean peak bone mass (average of young, healthy adults) as measured by dual-energy X-ray absorptiometry [1-6]. Osteoporosis can be further classified as primary, secondary, or idiopathic. People over the age of 70 years old and postmenopausal women develop primary osteoporosis, while secondary osteoporosis is typically caused by diseases and treatments such as metabolic bone disease and chronic corticosteroid use, respectively [6-8].

The most common clinical manifestation of osteoporosis is fractures, particularly in the vertebral bodies, ribs, proximal femurs, humeri, and distal radiuses, usually following a fall [6]. These fractures can result in chronic pain, loss of mobility, and decreased quality of life. Vertebral compression fractures can also lead to severe complications such as loss of height, kyphosis, postural changes, and chronic lower back pain [6,9]. Additionally, these fractures are associated with increased healthcare costs due to evaluations, surgical interventions, and in some instances, hospitalizations [10]. Budhia et al. reported in the year following an osteoporotic fracture, medical and hospitalization costs were 1.6-6.2 higher than pre-fracture costs and 2.2-3.5 times higher than those for matched controls [10].

Previous studies have described risk factors that increase the chance of developing osteoporosis, and they are broken down into non-modifiable and modifiable risk factors [3,8]. Non-modifiable risk factors for osteoporosis include positive family history, white ethnicity, female gender, and increased age, with the highest prevalence seen in postmenopausal women [3,8]. Lifestyle habits, including low body weight (BMI < 21 kg/m^2^), smoking, increased alcohol consumption, lack of exercise, long-term use of corticosteroids, and deficient calcium intake through diet, are modifiable risk factors for osteoporosis [3,8]. 

The Rio Grande Valley (RGV) in South Texas has a 90% Hispanic population that is severely medically underserved and faces many socioeconomic challenges that can contribute to increased risk for osteoporosis [11,12]. Sharkey et al. determined that only 28% of children in the RGV consumed the recommended daily amount of calcium, while none met the recommended amount of vitamin D [13]. 

Previous studies have shown a disproportionately high incidence of various diseases in the RGV compared to the national average, such as type 2 diabetes mellitus, Kaposi sarcoma, cervical cancer, and Alzheimer’s Disease [11,12,14,15]. Yet, to our knowledge, little is known about the incidence of osteoporosis, or screening for osteoporosis, in the RGV. Therefore, we aimed to gain insights into the osteoporosis landscape in the RGV compared to the broader national context. This will allow us to identify potential disparities in osteoporosis prevalence and screening practices, which can inform targeted healthcare interventions and public health strategies. We hypothesize individuals residing in the RGV have higher rates of osteoporosis and lower rates of osteoporosis screening than the national average. Of note, this article was previously presented as a meeting poster at the 2023 University of Texas Rio Grande Valley School of Medicine Research Colloquium on February 24, 2023.

## Materials and methods

This study involved the secondary analysis of publicly available data and, as such, did not require Institutional Review Board approval. We utilized the "Mapping Medicare Disparities by Population" interactive tool on "Data.CMS.gov" to retrospectively review Medicare beneficiary data. Our analysis focused on osteoporosis data and aimed to compare osteoporosis-related metrics within the RGV (comprising Starr, Hidalgo, Cameron, and Willacy counties) and nationally from 2016 to 2021.

We obtained various osteoporosis-related indicators from the "Mapping Medicare Disparities by Population" interactive tool. These indicators encompassed osteoporosis screening rates, overall osteoporosis prevalence, ethnicity-stratified osteoporosis prevalence, and sex-stratified osteoporosis prevalence. The interactive tool focused on the Medicare fee-for-service population, spanning 2016 to 2021. The measures included prevalence, adjustment smoothed actual data, analysis based on the measure, and differences from the national average, with the primary domain being chronic conditions, specifically osteoporosis. Sex categories were delineated as all, female, and male for all counties, with inclusion across all age categories. The racial and ethnic breakdown comprised white and Hispanic individuals, as data for Black, Asian/Pacific Islander, and American Indian/Alaskan Native populations were limited for the RGV region. All individuals under the Medicare eligibility category were encompassed in the study. Other potential risk factors contributing to osteoporosis, such as age, smoking status, drinking habits, prior fractures, or corticosteroid use, could not be differentiated due to the lack of specificity in the database regarding the presence or absence of these factors.

All rates were standardized, expressed as rates per 10,000 Medicare beneficiaries. Various statistical methods were applied to assess disparities and differences in osteoporosis prevalence and screening rates. Prevalence ratios were calculated by dividing rates to gauge the relative risk of osteoporosis in the RGV region compared to national averages. We presented 95% confidence intervals to evaluate the precision of our estimates. Chi-square values were utilized when applicable for statistical analysis to determine statistical significance.

## Results

Screening rates

**Figure 1:**
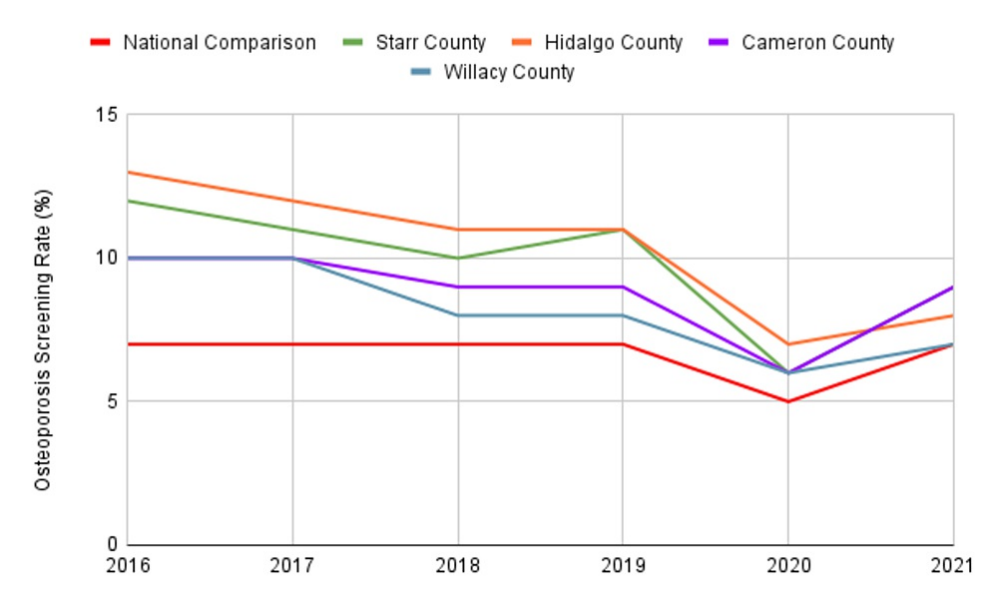
Osteoporosis screening rates (%) in the Rio Grande Valley compared to the US national mean from 2016 to 2021 as reported by CMS CMS: Centers for Medicare and Medicaid Services

Between 2016 and 2021, osteoporosis screening rates in the RGV consistently exceeded the national average, with a substantial difference (9.3% vs. 6.7%, p < .00001, 95% CI (1.10, 1.68)). Figure 1 visually represents each county's osteoporosis screening rates over the past six years, comparing them to the national average.

Overall osteoporosis prevalence

**Figure 2:**
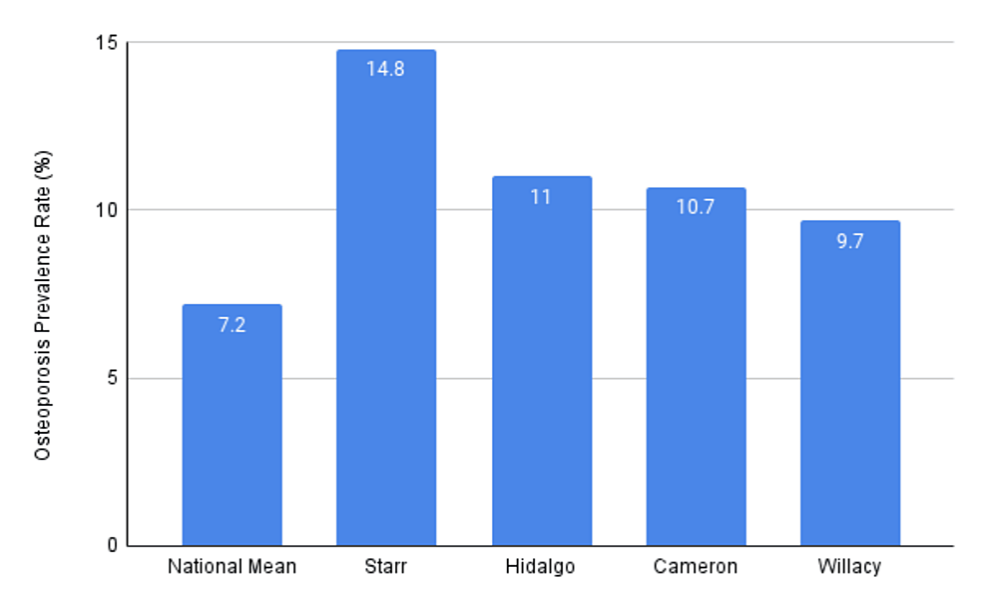
Average osteoporosis rates (%) in Rio Grande Valley counties compared to the US national mean from 2016 to 2021 as reported by CMS CMS: Center for Medicare and Medicaid Services

The overall rate of osteoporosis among RGV residents (11.5%) significantly surpassed the national average (7.2%) (p < .00001, 95% CI (1.41, 1.79)). Individual county rates are illustrated in Figure 2, with Starr County exhibiting the highest prevalence (14.8%). The remaining RGV counties, Hidalgo (11%), Cameron (10.7%), and Willacy (9.7%), also recorded higher rates compared to the national average (p <.0001 for all counties).

Osteoporosis by sex

**Figure 3:**
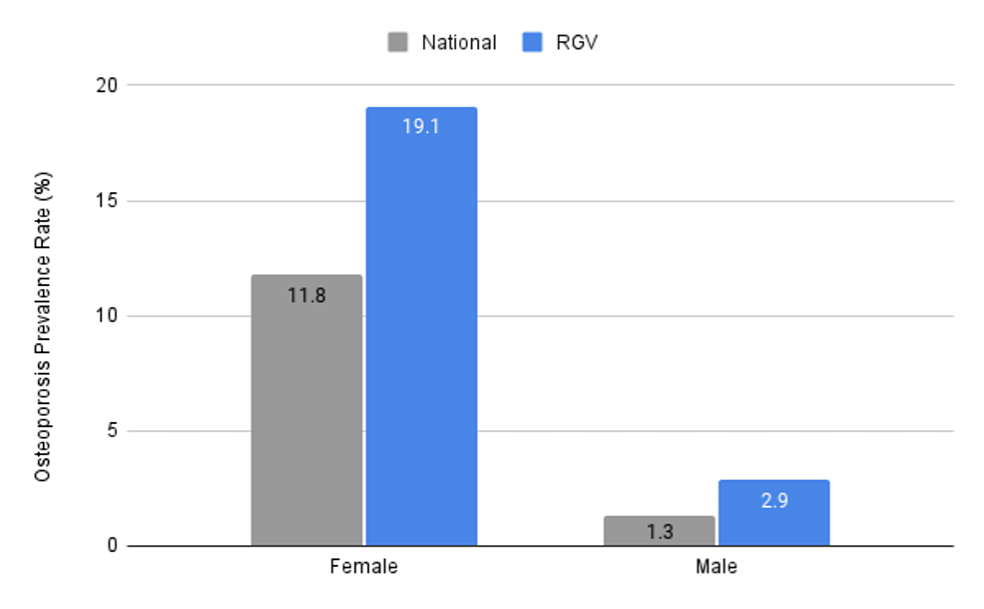
Average rates (%) of osteoporosis by sex nationally and in the RGV (Starr, Hidalgo, Cameron, Willacy - averaged) from 2016 to 2021 as reported by CMS CMS: Center for Medicare and Medicaid Services; RGV: Rio Grande Valley

Nationally, female Medicare beneficiaries had an overall osteoporosis prevalence of 11.8%. In the RGV, this rate was nearly double the national average, at 19.1% compared to 11.8% (p < .00001, 95% CI (1.20, 1.66)). Notably, RGV females (19.1%) exhibited a significantly higher prevalence of osteoporosis compared to RGV males (2.9%), marking a 6.58-fold increase (p < .00001, 95% CI (6.54, 6.64)). Males in the RGV were more than twice as likely to have osteoporosis compared to the national rate (2.9% vs. 1.3%, p < .00001, 95% CI (1.98, 2.48)). Figure 3 illustrates the prevalence of osteoporosis based on sex within the RGV compared to the national average.

Osteoporosis by ethnicity

**Figure 4:**
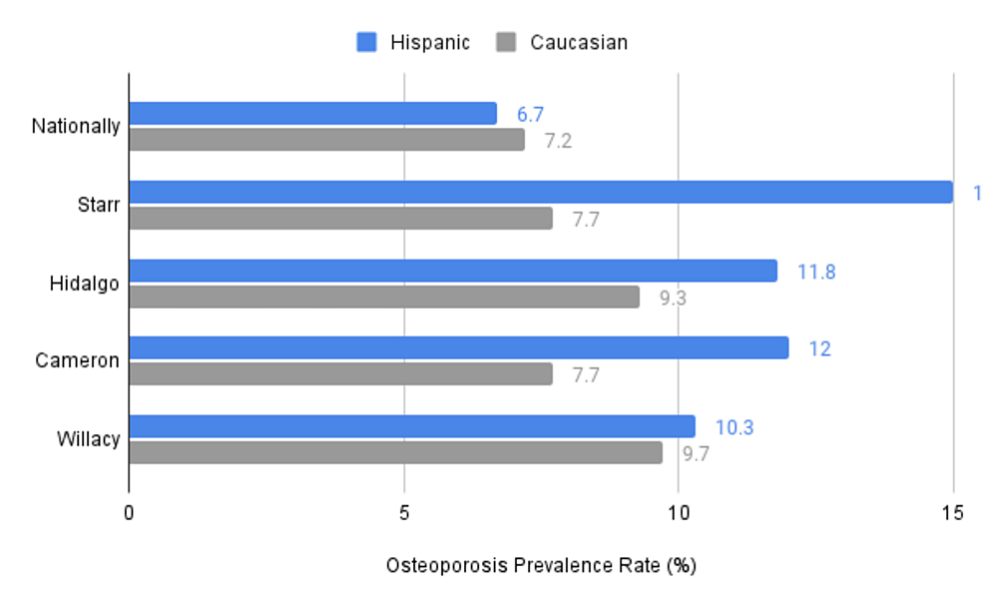
Osteoporosis rates (%) in the Rio Grande Valley based on ethnicity comparing Hispanics vs. Caucasians from 2016 to 2021 as reported by CMS CMS: Center for Medicare and Medicaid Services

In the RGV, the two most prevalent ethnic groups are Hispanics and Caucasians. Hispanics consistently displayed higher rates of osteoporosis than Caucasians across all counties. When averaging osteoporosis rates in the RGV, Hispanics had a prevalence of 12.3%, while Caucasians had 8.60% (p < .00001, 95% CI (1.20, 1.66)). Both ethnic groups in all counties had higher rates than the national mean (see Figure 4). Notably, the most significant discrepancy was observed among Hispanics in Starr County, with a prevalence rate of 15%. In this county, Hispanics had nearly double the rate of osteoporosis compared to Caucasians (15% vs. 7.7%, p < .00001, 95% CI (1.20, 1.66)). Other ethnicities within the "Medicare Mapping Medicare Disparities by Population" interactive tool included Black, Asian/Pacific Islander, and American Indian/Alaska Native; however, these results were unavailable due to limited Medicare beneficiaries with such ethnicities residing in the RGV.

## Discussion

There are no recent reports on how the RGV is impacted by osteoporosis; however, this is a region with known healthcare disparities and low funding [16,17]. Therefore, we aimed to gain further knowledge regarding the prevalence of osteoporosis within the RGV compared to the broader national context. To our knowledge, our study is the first to report on the healthcare disparities surrounding osteoporosis, specifically within the RGV. Our data suggests that patients residing within the RGV, irrespective of ethnicity, are disproportionately impacted by osteoporosis when compared to the rest of the nation.

Osteoporosis poses a significant health concern for Hispanics residing in South Texas, influenced by a multitude of factors such as low education levels, socioeconomic status, genetics, lifestyle choices, and cultural nuances. The RGV symbolizes this scenario, characterized by a generally low socioeconomic status and a substantial Mexican-American population, amplifying the health risks in the region [18]. Although limited by not having an individual-level data population, investigating whether individuals in lower-income brackets face a higher risk of osteoporosis could provide valuable insights into the complex interplay of socioeconomic factors and bone health in the region. Despite this, the genetic predisposition of Hispanics to have lower BMD than non-Hispanic whites contributes to an elevated susceptibility to osteoporosis [19]. Furthermore, lifestyle factors, including sedentary habits, smoking, and excessive alcohol consumption, are prevalent among Hispanics in South Texas, accentuating the risk of osteoporosis [17,20]. 

Cultural factors, notably adherence to traditional Hispanic diets, contribute to the osteoporosis risk within this population. These dietary patterns, potentially lacking in essential bone-healthy elements like calcium, vitamin D, and zinc, may contribute to an increased susceptibility to osteoporosis [1,21]. Furthermore, it is noteworthy that the RGV exhibits some of the highest obesity levels in the nation, theoretically providing a protective effect against osteoporosis [2,20]. However, despite the elevated rates of obesity, a significant health disparity persists, highlighting the complex interplay of multifactorial risk factors in bone health and osteoporosis. Therefore, comprehending and addressing the multifaceted influences, alongside potential confounding variables such as genetics, diet, activity levels, and the presence or absence of menopause, is crucial for formulating effective preventive strategies.

Current literature underscores the importance of variability of BMD and osteoporosis rates within specific racial and ethnic groups, emphasizing the need for additional research in individual groups based on origin or background [3,22]. Our study reinforces this perspective by revealing a higher prevalence of osteoporosis (12.3%) among the Hispanic population in contrast to non-Hispanic whites (8.6%). Furthermore, our data sheds light on the disproportionate impact of osteoporosis in the RGV, where the mean osteoporosis rate stands at 11.5%, surpassing the national average of 7.2% despite an increased screening rate (9.3% vs. 6.7%). Moreover, the region is also impacted by gender-specific patterns of osteoporosis prevalence at higher rates compared to the national average in both females and males, even though male sex is typically considered protective against osteoporosis (19.1% vs. 11.8% and 2.9% vs. 1.3% in the RGV vs. nationally, respectively). Although consistent with current literature, these findings emphasize the gendered nature of osteoporosis risk and highlight the need for gender-specific approaches for osteoporosis education and treatment [1-3,23].

This study has limitations. First, this retrospective observational study is based on Medicare beneficiary data; therefore, the results may not be generalizable to all residents of the RGV. These results may not accurately reflect, and even underreport, the rates of osteoporosis, given the disproportionate amount of residents who are illegal immigrants, indigent, or do not have access to healthcare insurance to seek appropriate screening [17,24]. While screening rates were elevated among residents with Medicare benefits in the RGV, it remains uncertain how many of these individuals sought treatment for osteopenia (before developing osteoporosis), considering the region's restricted access to healthcare [24]. Additionally, we could not stratify results for individual-level data, account for osteopenia prevalence, and had no way to account for any confounding risk factors given the database. Further research should consider utilizing hospital-acquired data and community screening event data to further enhance our understanding of the applicability of our findings. This would significantly bolster the generalizability of our results and could serve as a valuable reference for how we can monitor and treat those impacted by osteoporosis.

Moreover, it’s important to note that our study did not report on all ethnicities as the "Mapping Medicare Disparities by Population" interactive tool had insufficient data for Black, Asian, and Native American residents residing in the RGV. Existing literature has demonstrated an increased risk of osteoporosis in Asians and Native Americans, and it is unclear if this would heighten the disparity noted within the region [25]. The RGV is primarily composed of Hispanics, making up approximately 90% of the region's residents; therefore, this could have also led to skewed rates of osteoporosis [12]. However, despite these findings, Caucasians in the RGV were also found to be disproportionately impacted by osteoporosis when compared to national prevalence rates.

## Conclusions

This study is the first to compare the rates of osteoporosis in the RGV against the national average, revealing a significant and concerning disparity. Despite elevated screening rates in the region, the prevalence of osteoporosis in the RGV disproportionately exceeds the national average, highlighting a distinctive healthcare disparity. Hispanic women residing in the RGV, in particular, face a heightened vulnerability to osteoporosis compared to Caucasians. We believe this is largely due to a complex interplay of genetic, lifestyle, and cultural factors contributing to susceptibility. Combating this health inequity will require improving healthcare accessibility in the region’s lower socioeconomic areas and a concerted effort to raise awareness and implement targeted prevention strategies. Furthermore, through the insights gathered in this paper, we hope this provides an opportunity to tailor healthcare interventions and public health strategies to meet the unique needs of the RGV community.

## References

[1] Aibar-Almazán A, Voltes-Martínez A, Castellote-Caballero Y, Afanador-Restrepo DF, Carcelén-Fraile MD, López-Ruiz E (2022). Current status of the diagnosis and management of osteoporosis. Int J Mol Sci.

[2] Armas LA, Recker RR (2012). Pathophysiology of osteoporosis: new mechanistic insights. Endocrinol Metab Clin North Am.

[3] Kelsey JL (1989). Risk factors for osteoporosis and associated fractures. Public Health Rep.

[4] Lane JM, Russell L, Khan SN (2000). Osteoporosis. Clin Orthop Relat Res.

[5] Rachner TD, Khosla S, Hofbauer LC (2011). Osteoporosis: now and the future. Lancet.

[6] Glaser DL, Kaplan FS (1997). Osteoporosis. Definition and clinical presentation. Spine (Phila Pa 1976).

[7] Marcucci G, Brandi ML (2015). Rare causes of osteoporosis. Clin Cases Miner Bone Metab.

[8] Salari N, Ghasemi H, Mohammadi L, Behzadi MH, Rabieenia E, Shohaimi S, Mohammadi M (2021). The global prevalence of osteoporosis in the world: a comprehensive systematic review and meta-analysis. J Orthop Surg Res.

[9] Becker DJ, Kilgore ML, Morrisey MA (2010). The societal burden of osteoporosis. Curr Rheumatol Rep.

[10] Budhia S, Mikyas Y, Tang M, Badamgarav E (2012). Osteoporotic fractures: a systematic review of U.S. healthcare costs and resource utilization. Pharmacoeconomics.

[11] Bowden VM, Wood FB, Warner DG, Olney CA, Olivier ER, Siegel ER (2006). Health information Hispanic outreach in the Texas Lower Rio Grande Valley. J Med Libr Assoc.

[12] Innis-Whitehouse W, Wang X, Restrepo N, Salas C, Moreno K, Restrepo A, Keniry M (2018). Kaposi sarcoma incidence in females is nearly four-fold higher in the Lower Rio Grande Valley compared to the Texas average. Cancer Treat Res Commun.

[13] Sharkey JR, Nalty C, Johnson CM, Dean WR (2012). Children's very low food security is associated with increased dietary intakes in energy, fat, and added sugar among Mexican-origin children (6-11 y) in Texas border Colonias. BMC Pediatr.

[14] Garza N, Uscamayta-Ayvar M, Maestre GE (2020). Addressing neurocognitive disorders, dementias, and Alzheimer’s disease in colonias of the lower Rio Grande Valley: establishing a research foundation using promotores. Ethn Dis.

[15] Salcedo MP, Gowen R, Rodriguez AM (2023). Addressing high cervical cancer rates in the Rio Grande Valley along the Texas-Mexico border: a community-based initiative focused on education, patient navigation, and medical provider training/telementoring. Perspect Public Health.

[16] Logan RI, Castañeda H (2020). Addressing health disparities in the rural United States: advocacy as caregiving among community health workers and promotores de Salud. Int J Environ Res Public Health.

[17] Ramirez AG, Thompson IM, Vela L (2013). The South Texas Health Status Review: A Health Disparities Roadmap.

[18] Diego VP, Manusov EG, Mao X (2023). Genotype-by-socioeconomic status interaction influences heart disease risk scores and carotid artery thickness in Mexican Americans: the predominant role of education in comparison to household income and socioeconomic index. Front Genet.

[19] Looker AC, Melton LJ 3rd, Harris T, Borrud L, Shepherd J, McGowan J (2009). Age, gender, and race/ethnic differences in total body and subregional bone density. Osteoporos Int.

[20] Larsen BA, Pekmezi D, Marquez B, Benitez TJ, Marcus BH (2013). Physical activity in Latinas: social and environmental influences. Womens Health (Lond).

[21] Föger-Samwald U, Dovjak P, Azizi-Semrad U, Kerschan-Schindl K, Pietschmann P (2020). Osteoporosis: pathophysiology and therapeutic options. EXCLI J.

[22] Noel SE, Santos MP, Wright NC (2021). Racial and ethnic disparities in bone health and outcomes in the United States. J Bone Miner Res.

[23] De Martinis M, Sirufo MM, Polsinelli M, Placidi G, Di Silvestre D, Ginaldi L (2021). Gender differences in osteoporosis: a single-center observational study. World J Mens Health.

[24] Kuruvilla R, Raghavan R (2014). Health care for undocumented immigrants in Texas: past, present, and future. Tex Med.

[25] Barrett-Connor E, Siris ES, Wehren LE (2005). Osteoporosis and fracture risk in women of different ethnic groups. J Bone Miner Res.

